# 被误诊为Coombs阴性自身免疫性溶血性贫血的维生素B_12_假性正常的巨幼细胞性贫血1例

**DOI:** 10.3760/cma.j.cn121090-20240719-00270

**Published:** 2024-11

**Authors:** 炳臣 朱, 显勇 蒋, 炎 张, 苗 陈, 为 王

**Affiliations:** 1 中国医学科学院北京协和医院血液内科，北京 100730 Department of Hematology, Peking Union Medical College Hospital, Chinese Academy of Medical Sciences, Beijing 100730, China; 2 中国医学科学院北京协和医院全科医学科，北京 100730 Department of General Internal Medicine, Peking Union Medical College Hospital, Chinese Academy of Medical Sciences, Beijing 100730, China

患者，男，36岁，因头晕、面色苍白1个月余，于2024年4月26日入北京协和医院。患者2024年3月17日新型冠状病毒感染发热后逐渐出现头晕，进行性加重，伴尿色加深、面色苍白、全身乏力，3月27日于外院查外周血：RBC 1.90×10^12^/L、HGB 76 g/L、WBC 3.31×10^9^/L、PLT 144×10^9^/L、平均红细胞体积（MCV）110.0 fl；总胆红素（TBil）38.9 µmol/L、直接胆红素（DBil）9.2 µmol/L、LDH 3 088 U/L；血维生素B_12_（Vit B_12_）、叶酸正常；直接抗人球蛋白试验（Coombs试验）阴性；补体C3 642 mg/L、免疫球蛋白、抗核抗体谱、抗中性粒细胞胞质抗体（ANCA）、类风湿抗体谱、类风湿因子均阴性；红细胞渗透性试验阴性；骨髓象：增生极度活跃，粒红比为0.77∶1，红系增生显著，原始阶段的巨幼红细胞可见，早期阶段的巨幼红细胞易见，中期、晚期阶段的巨幼红细胞多见，可见分裂象、花斑样晚期阶段巨幼红细胞、点彩晚期阶段巨幼红细胞、豪焦（Howell-Jolly）小体，成熟红细胞大小不等，部分胞体大，可见异型红细胞碎片（红系病态造血占48％），血小板减少；检查诊断：骨髓增生异常综合征（MDS）不排除；进一步完善骨髓免疫分型未见异常表型，血液病相关基因突变及融合基因、MDS-FISH、骨髓细胞组化染色、阵发性睡眠性血红蛋白尿（PNH）克隆均未见明显异常；腹部、盆腔CT：脾大，幽门区可见类圆形囊性低密度灶，长径约18 mm。2024年4月10日于外院查外周血：HGB 55 g/L；外院考虑Coombs阴性的自身免疫性溶血性贫血（AIHA）可能性大，予以泼尼松（50 mg/d，每日1次，口服），患者自觉症状未见明显好转，复查HGB 59 g/L。患者起病以来，活动耐量较前下降，间断茶色尿，近期出现双手、双脚麻木，感觉对称，步态平稳，无脚踩棉花感，食欲下降，睡眠可，近2年反复出现口腔溃疡，否认外阴溃疡。既往史：慢性支气管炎、过敏性鼻炎6年余，春冬季节易发作；白癜风5年余；个人史、家族史无特殊。入院体格检查：生命体征平稳，全身皮肤黏膜可见散在白色斑片，贫血貌，舌体光滑、苍白，呈“牛肉样舌”，脾大，约肋缘下2 cm；双下肢未见水肿。2024年4月26日就诊我院查外周血：RBC 1.28×10^12^/L、HGB 52 g/L、MCV 113.3 fl、PLT 120×10^9^/L、WBC 7.91×10^9^/L、TBil 38.6 µmol/L、DBil 13.8 µmol/L，血Vit B_12_ 205 pg/ml、叶酸11.8 ng/ml、网织红细胞绝对值73×10^9^/L；内因子抗体（IFAb）126.98 AU/ml；同型半胱氨酸（HCY）59.6 µmol/L；抗胃壁细胞抗体（a-PCA）阳性，滴度为1∶160。外周血涂片：红细胞大小不等，可见大红细胞，中性分叶核粒细胞可见多分叶现象；脾脏彩超：脾厚径3.5 cm、长径17.5 cm、肋缘下1.9 cm。

患者存在经典“牛肉样舌”表现，MCV明显增高，血涂片中性粒细胞多分叶，骨髓形态可见红细胞系巨幼细胞性变化，IFAb阳性，虽然Vit B_12_正常，但仍诊断考虑巨幼细胞性贫血，其Vit B_12_正常考虑为检测假性正常。2024年4月27日起予以VitB_12_，具体为Vit B_12_ 500 µg，每日肌注1次，1周；之后改为Vit B_12_ 500 µg每周肌注1次，1个月；最后Vit B_12_ 500 µg每月肌注1次维持，未联合其他药物治疗。2024年5月28日查RBC 4.16×10^12^/L、HGB 126 g/L、MCV 92.3 fl、PLT 219×10^9^/L、WBC 4.2×10^9^/L、TBil 11.5 µmol/L、DBil 4.3 µmol/L，贫血貌消失，双手双脚麻木较前好转，未再发头晕。HGB恢复正常后完善胃镜检查，检查诊断：慢性萎缩性胃炎（胃底体为主），自身免疫性胃炎可能；病理结果：胃体胃黏膜活检：中度慢性炎伴重度肠化；胃底胃黏膜活检：重度慢性炎伴中度肠化。2024年6月11日电话随访，患者未再出现头晕、双手双足麻木。

